# The experience of conscious reflection within a general theory of learning

**DOI:** 10.3389/fpsyg.2025.1587455

**Published:** 2025-06-18

**Authors:** Espen Hoff Dyngeland

**Affiliations:** Department of Sport, Food and Natural Sciences, Faculty of Education, Arts and Sports, Western Norway University of Applied Sciences, Bergen, Norway

**Keywords:** flow theory, learning, negation, phenomenology, reflection, equilibration

## Abstract

One of the most important constructs in educational research and practice is learning. Yet, it is also one of its most complicated, ambiguous, and debated constructs. Questions regarding what constitutes the process of learning and how best to facilitate it have been addressed from numerous perspectives, often yielding competing interpretations and approaches. One aspect that is often the subject of these debates is the extent to which learning is an actively conscious activity. This study will contribute with a conceptual dialogue between different strands of scholarship that attend to the role of consciousness and the experience of learning, as well as those cognitive processes theorized to underlie these experiences. The upshot of this dialogue will be to connect the different theoretical perspectives, resulting in a wider viable conceptual apparatus to describe the experience of learning, founded in theoretical perspectives that seek to illuminate the cognitive processes that underlie these experiences.

## Introduction

Theories pertaining to the processes of learning and how to facilitate such processes are widely varied and interdisciplinary. No matter which theoretical perspective one may adhere to, the extent to which learning is a conscious and reflective process concerns all aspects of learning. However, while the facilitation of the process of learning, as well as what constitutes learning in general, is often discussed, less is known concerning how the process of learning is experienced from the first-person perspective. In this study, I will engage in a conceptual dialogue between three fields of scholarship: (1) contemporary approaches to the paradigm of constructivist learning theories, (2) *flow-theory,* and (3) phenomenological perspectives on *learning as negation*. The aim of the ensuing theoretical discussion is to enrich the conceptual frameworks within constructivist learning theories with perspectives on the experiences of learning with two contrasting theoretical frameworks.

### Steps toward a general theory of learning

To lay the groundwork for this discussion, we must first briefly consider the influential paradigm of contemporary approaches to constructivist learning theories^1^ that aim to conceptualize a general theory of human learning. Roughly construed, this strand of scholarship conceptualizes learning as a dynamic, situated, and socially mediated process through which individuals actively construct knowledge. Rather than treating knowledge as a static set of facts to be transmitted, constructivism posits that knowledge emerges through the interaction between knowers, situations that require knowledge, and peers. This interaction is fundamentally relational and recursive, meaning that it is shaped by learners’ existing cognitive structures and, when need be, transformations of those structures through the process of learning. As a starting point, Piaget (1952, 2005) argued that subjects are inclined to maintain a balance, or organizational stability (cf. Di Paolo et al., 2014), between what they know and the experiences they have in meetings with the world and other subjects. However, human beings continuously face situations that are completely new to them, and therefore, this system is inherently responsive and dynamic. To account for these new inputs, Piaget famously conceptualized the processes of assimilation and accommodation, together termed the process of equilibration. In brief terms, assimilation refers to the process of integrating knowledge into already existing knowledge structures or cognitive schemas. Accommodation, on the other hand, is necessary when new information does not align with existing schemas, leading to a state of disequilibrium. If the subject becomes aware of this imbalance, it may result in an enduring cognitive conflict (cf. Sánchez and Sebastián, 2024; Sebastián et al., 2021, p. 239), necessitating the formation of a new or transformation of current knowledge structures to maintain equilibrium (Piaget, 1985).

Accepting these general principles also entails acknowledging that learning does not come about in a vacuum. Rather, for the process of learning to occur, some sort of provocation is necessary (Mezirow, 1991). In other words, the cognitive conflict must in some shape or form be experienced as socially or biographically meaningful (Bourgeois, 2011), if it is to spark a sense of motivation for the process of learning.

In this vein, Sánchez and Sebastián (2024) further develop the conceptualisation of cognitive conflict with Vygotsky and Cole’s (1978) general principles pertaining to the zone of proximal development in mind. They state that the cognitive conflict must remain within (…) *the limits of previous knowledge structures so that they can be deconstructed and socially reconstructed into something truly new, thus generating a progressive advance and active construction of knowledge* (p. 241), for the process of learning to occur.

By transcending what they term as methodological individualism of transformative learning theories, Sebastián et al. (2021) state that the genesis of novelty (new knowledge) is dependent on a relational-dialectical approach and can be explained by the interactions between the subject and its environment. To support this view, they turn to Vygotskij’s concept of internalization, which entails a “structural and functional change of the entire operation as a whole” (Vygotski and Luria, 2007, p. 66, cited in Sebastián et al., 2021). Internalization refers to the process by which inputs, initially of external origin (such as conversations with others), become internalized and integrated into a subject’s cognitive structures. While internalization refers to similar processes as those sketched out by Sebastián et al. (2021) contend that Vygotskij’s account is better suited for explaining the problem of novelty, that is, how completely new knowledge can come to be. This is particularly due to his concept of the zone of proximal development, which distinguishes the processes that happen on the intrapsychic plane from those on the interpsychic plane, which in turn can function as the foundation for novelty.

In summary, Sebastián and colleagues sketch out a comprehensive model that delineates the relationship between the processes formulated by the two scholars: In general terms, subjects interact with the world and people around them in a relatively stable fashion due to the cognitive processes that allow objects of knowledge to be assimilated without further complication, so to speak. However, considering those mentioned above, certain situations can lead to enduring states of cognitive conflicts. To again return to equilibrium, a restructuration by a Vygoskijan internalization constitutes the process of learning (Sebastián et al., 2021). Thereby, learning is not instantaneous but unfolds as learners take positions within and respond to the tensions of their evolving participation in meeting with the world and other subjects [Sebastián and Lissi, 2016, cited in Sánchez and Sebastián (2024)].

Considering the argument that learners need to be aware of the disequilibrium, the concept of (conscious) reflection appears relevant. One often-cited source of inspiration for reflection in the context of learning stems from the experiential learning theory formulated by Kolb (2014). For Kolb, reflection served as the key for learners to transform experiences into knowledge, and it can thereby be termed a fundamental aspect of the process of learning. According to Agouridas and Race (2007), reflection involves personalizing and making sense of what subjects have learned by linking their experiences to broader perspectives and understanding the underlying rationale. Furthermore, reflection has also been argued as being an avenue toward tapping into prereflective or tacit knowledge (Helyer, 2015), as well as inherently situated in experience and sociality (Ovens and Tinning, 2009). The latter point on sociality is important, as research has underpinned that reflection is not sufficient in and of itself to thwart discouragement (Mälkki and Lindblom-Ylänne, 2012), which aligns with the idea that cognitive conflicts must be experienced as meaningful. Returning to the strides made within the constructivist paradigm, Sánchez and Sebastián (2024) frame reflection as (1) the process of becoming aware of experiences of doubt and uncertainty and (2) “*as a selfregulatory movement, consistent with the emergence and unveiling of conflict in the equilibration process*” (p. 243).

As evident through these introductory remarks on the learning process, the cognitive processes that underpin the process of learning are dedicated to an impressive body of literature. However, many of these investigations into the processes of learning often remain abstract and far removed from the experiential dimensions from the perspective of the learner. While Sánchez and Sebastián (2024) have made strides toward investigating the role of emotions and affectivity within the bounds of their general theory of learning, little is still known about the conceptual interplay between theories that pertain to the cognitive process of learning and those that pertain to the experience of the learning process from the learners perspective.

My aim in this paper, therefore, is to utilize flow theory and learning as negation as perspectives that will contribute to addressing the qualitative experience in moments of (1) assimilation, (2) disequilibrium and cognitive conflict, and (3) accommodation and reflection. In what follows, I will present the two aforementioned frameworks that contribute to understanding how learners’ consciousness is suspended in a buoyant state between immersion and reflection, followed by situating these insights within the processes outlined by constructivist theories.

## Flow theory

Flow was developed by Mihaly Csikszentmihalyi and can be described as a state human beings can be encompassed by when engaging in activities (Csikszentmihalyi, 2000). It has been widely researched empirically through the perspectives of people with significant expertise within a particular field, such as talented musicians (Harmat et al., 2021), dancers (Łucznik and May, 2021; Panebianco-Warrens, 2014), or professional athletes (Özdemir and Durhan, 2020). Furthermore, it has also been adapted and utilized as a concept within education, both empirically and conceptually (Csikszentmihalyi, 2014a). Flow has been transposed into educational settings under the promise of engaging students in a time where “student engagement” has seen a huge rise in interest (Bond et al., 2020). The rise of interest in student engagement is justified, seeing that research indicates that it may have impacts on academic achievement (Reyes et al., 2012) and school completion (Archambault et al., 2009). Against this background, the promise of flow is highly enticing for educators, policymakers, and researchers alike.

Csikszentmihalyi conceptualizes an experience of flow through nine different conditions: (1) The goals of the activity one is engaging in are clear; (2) Feedback within the given situation is immediate, meaning that the subject is constantly aware that the performance in the given task or activity is sufficient; (3) The skill of the subject matches the challenges at hand, meaning that there is a sense of balance between expectations and reality; (4) The subject is in a deep, attentive, and concentrated state; (5) Problems and irrelevant stimuli are excluded from consciousness; (6) Success within the given task lies in principle solely with the subject itself, few to no external factors determine the outcome; (7) Self-consciousness disappears, which Csikszentmihalyi states is the transcending of one’s ego; (8) Time is experienced as passing much faster than usual; (9) The experience is autotelic, meaning it is worth engaging in for its own sake (i.e., not extrinsically motivated) (Csikszentmihalyi, 2014b).

As one can deduct from these conditions, the facilitation of flow is a difficult process, seeing that many of the conditions cannot be regulated by an outsider. Rather, the subject engaging in the specific act is the one who sets the preconditions and boundaries for a state of flow to occur. In the same vein, the conditions are also different in form. Some conditions, for example, 1, 2, 3, and 6, relate specifically to the task or activity at hand and can, as such, be termed as object-specific conditions. The object-specific conditions are highly diverse. Contrast the situations of engaging in running, an activity where flow has been widely researched (Csikszentmihalyi et al., 2017), and a classroom setting. Running is (or at least can be) an activity in which the subject can freely negotiate the terms and conditions. The goals (condition 1, in the case of running: distance, pace, time, altitude gain, etc.), as well as how the subject relates to them, differ widely depending on whether one is engaging in running for leisure or as a professional athlete. However, for a pupil in a classroom setting, the subject itself does not have the same agency to determine the specific object-specific conditions as it would if they were engaging in an autotelic activity. As is well known, pupils hold widely different preconditions and competences prior to meeting the somewhat predetermined boundaries provided by the teacher and the curriculum (the goals, so to speak).

Conditions 4, 7, 8, and 9, on the other hand, are subject-specific conditions. What I mean by subject-specific conditions is that no matter what activity one engages in (be that running or a classroom setting), these factors remain static. If one were to be deemed within a state of flow, the subject-specific conditions would not differ depending on activity, as is the case with the object-specific ones. This has been alluded to in research within educational settings, where, e.g., Schmidt et al. (2014) comment on the conditions that I term as “object-specific” as being conditions of an “optimal experience,” while the conditions I term as subject-specific are typically present regardless of the activity.

**Figure 1:**
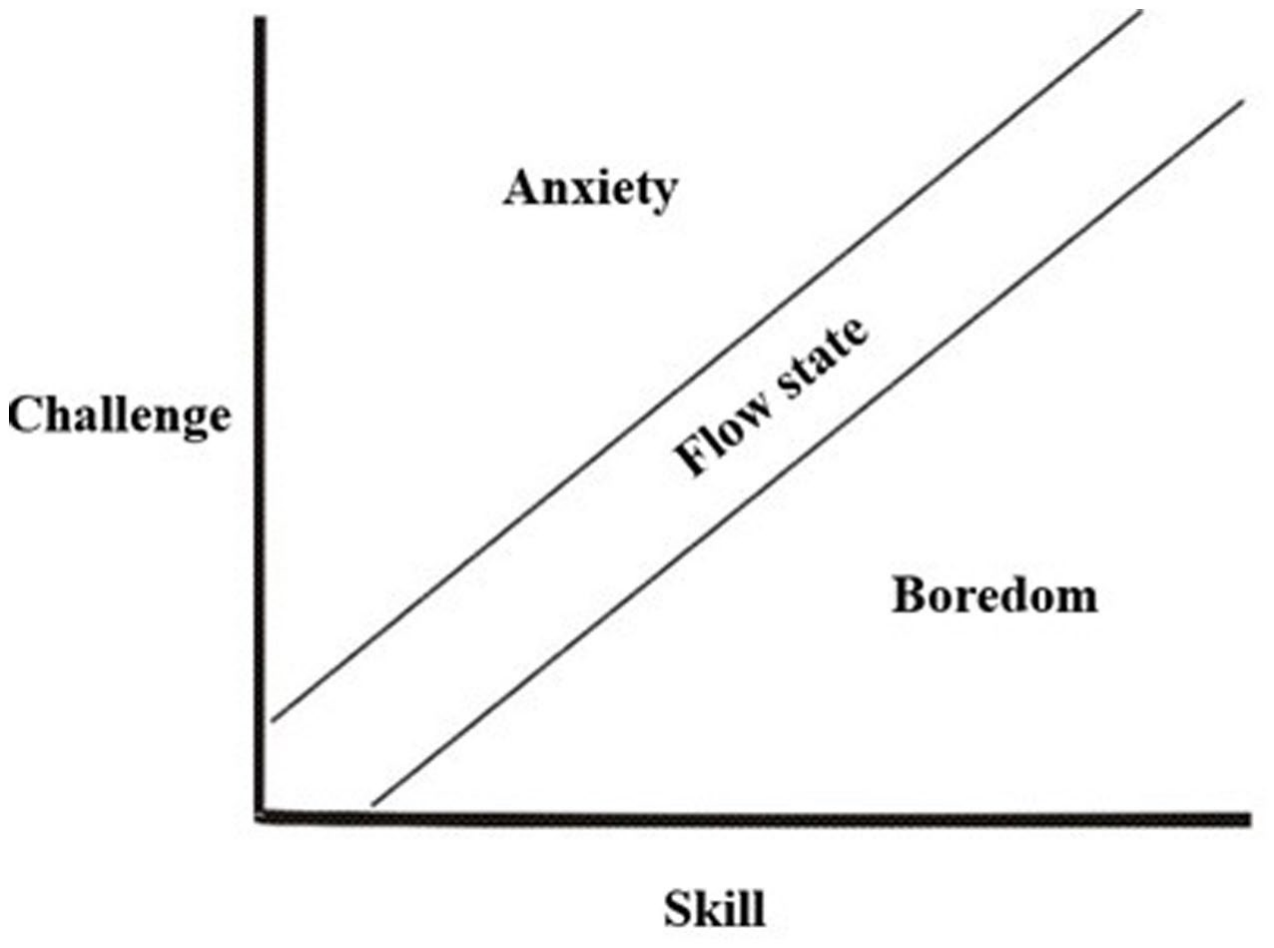
Flow—derived from Csikszentmihalyi (1990).

The following visualization is often provided in scholarly work on flow theory:

In summary, and as evident in Figure 1^2^, for the flow-state to occur, there must be a coherent relationship between the level of challenge the task at hand requires and the level of skill within that field that the subject already has (cf. condition 3). However, pupils in educational settings often face challenges that are completely new to them. Within flow theory, these situations would be characterized as placing the pupils in a state of anxiety. To further conceptualize the instances where this occurs, I will draw on the phenomenological approaches of Günther Buck, termed Learning as Negation.

### Learning as negation

Buck was a pedagogue who engaged with phenomenological philosophy and contributed considerably to education in the German context. His work *Lernen und Erfahrung* (Learning and Experience) has been regarded as a classic in German pedagogy since its first publication in 1967. For Buck, teaching and education always revolve around certain conceptualisations of learning, which for him was closely connected to experience (Brinkmann, 2016; Brinkmann and Friesen, 2018). However, Buck argues that learning, although being the pillar upon which education is based, is one of its most ambiguous concepts (Buck, 2019). In the newest edition of Buck’s work, Chapters 4 and 5 are dedicated (among other things) to the concept of *negation* and Husserl’s concept of intentionality, respectively. Now, to properly address Buck’s work on education, we first need to attend to Husserl’s analysis of inner-time consciousness.

Husserl’s well-known analysis of time consciousness has a triadic structure consisting of *retentions*, *primal impressions,* and *protentions* (Husserl, 2001, 2012). Retentions means holding in consciousness that which has just been experienced. Primal experiences are those sensory input which the subject is currently experiencing. Finally, protentions are the approximations or anticipation of what is to come. Husserl utilizes the example of music to illustrate this basic structure: Imagine listening to a melody, experiencing each note in the present moment; this is the primal impression, the immediate awareness of the now. As the melody unfolds, the subject retains a sense of the notes that have just been played, even though they are no longer present. This ongoing awareness of past notes is the retention, where past experiences linger and influence the subject’s current consciousness. Simultaneously, the subject anticipates the next note, expecting it to follow the established pattern of the melody—this anticipation is protention, where consciousness reaches forward into the future, predicting what is about to occur. These three components combine to create the flow of experience, which enables the subject to perceive the melody as a continuous and coherent sequence rather than as isolated sounds.

One can also distinguish between near protentions, which is analogous to the example of holding the previous tone of music in one’s consciousness, while expecting the next, and far protentions, which are of a more general and extended manner but not as determined (Rodemeyer, 2006). The far retentions can be conceptualized as being based on acquired habits or patterns throughout the life course. Fuchs (2022) illustrates far retentions by the example of spoken communication:

Similarly, when listening to spoken sentences, they often form extended braces, so that, for example, an “on the one hand” calls for an “on the other hand,” but this may occur with a considerable delay (p. 5).

**Figure 2:**
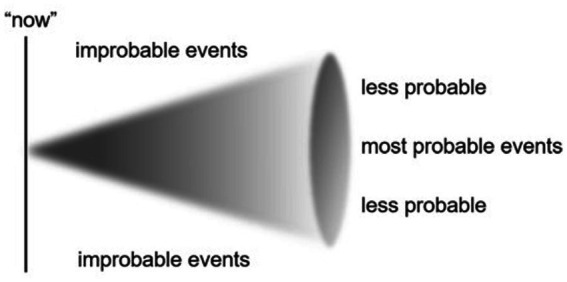
Protential cone (Fuchs, 2022).

Based on Husserl’s work, psychiatrist and phenomenologist Thomas Fuchs developed what he calls *the protential cone*, which provides a highly useful visualization of the retention-primal impression-protention structure:

The “now” in Figure 2 represents the primal impressions, and as deducible; the further the subject is removed (temporally) from the primal impression, the cone structure allows for a broader understanding of that which is “probable” (Fuchs, 2022).

With this basic understanding of Husserl’s triadic structure of temporal experience in mind, we can now consider how Buck draws on this framework in his conceptualisation of learning as negation. The structure of experiences of learning, on Buck’s account, involves cycles of anticipations (protentions) based on previously lived experiences (retentions) and ongoing primal impressions. These cycles can be cumulatively positive (i.e., fulfillment of the anticipation brought forth by the protention) or negative (i.e., negation of the protention). In this context, the concept of negation refers to the Hegelian *determinate negation*. Determinate negation was, for Hegel, a central facet of his dialectic method, which is a dynamic process wherein a concept’s or an idea’s limitations are acknowledged, so that new conceptualisations can emerge, allowing for a conceptual movement (Sparby, 2014). When Buck transposes the Hegelian determinate negation into educational settings, the lack of fulfillment of the protential cone represents a transformative experience (i.e., learning something new). When pupils experience a negation of a protention, they not only experience something external but also gain insight into themselves. Through this negation, their personal horizon (i.e., protential cone) shifts, and with it, their future expectations and perceptions of past events as well (Buck, 2019). As Brinkmann and Friesen (2018) put it:

The structure of experience as bound by a horizon is always based on previous experience, but it is also open to what is new or different—what can be delimited through its extension or expansion (Horizontwandel). As our horizon is changed in an experience, future anticipations change, as do our understandings of experiences from the past (p. 5).

Drawing on Buck, Meyer-Drawe (2018, 2019) termed this process as *Umlernen* (re-learning). Umlernen does not only include re-learning of something that was previously misunderstood but an alteration in the entire set of dispositions at hand for the learner (Buck, 2019). This implies that in the process of Umlernen, it is not only the ideas or content that undergo transformation but also the learner and their previous convictions. Thereby, conscious reflection remains a pillar within such approaches:

The beginnings of learning cannot be consciously self-initiated, rather they are felt in the pre-reflective experience of negativity, that is, of a break with ourselves, our habitual modes of being, our assumptions and our own learning history. These breaks or gaps in experience cannot simply be closed by attaining new knowledge, but they can be productively dealt with *through reflection and inquiry* (English, 2012, p. 212, emphasis added).

As evident, the process of Umlernen does not simply “go with the flow” of experience. Rather, as English puts it, such instances represent a break in experiences that can only be productively handled through active reflection. Still, the reflection does not have to be innately conscious, as it can come about in a split second of entanglement, experienced as a perplexing or surprising moment for the learner. Meyer-Drawe sees this moment of surprise as a moment wherein the learner experiences a “painful turn-around,” which concerns a shift in both prior knowledge and future probable situations (Meyer-Drawe, 2018, 2019). The breaks in experience, or painful turn-arounds, signified for Buck an experience wherein the learner is compelled to investigate, revise, and subsequently transform that which can be termed as irreconcilable contradictions between the ongoing primal impressions and the conceptual assumptions that the subject had previously operated with Buck (2019).

Having briefly outlined the three strands of scholarship this study engages with, the next section explores how their integration can offer a constructive heuristic for understanding the experience of learning grounded in the cognitive processes that underlie it.

### Conceptual interplay: the experience of learning

In brief terms, Flow, on the one hand, refers to an experience of intertwining and dilution between thematized self-awareness, activity at hand, and the passage of time. The subject becomes so engaged in the situation that the following course(s) of action are prereflectively experienced as natural and organic. Learning as negation, on the other hand, refers to situations where the very same anticipations (termed as protentions) that arise in the flow state appear, yet they do not present themselves as intuitive. Rather, they come about as a sense of foreignness or unknown, which provokes the activity of reflection (Buck, 2019). As such, the two frameworks present different conceptualizations of qualitatively lived experiences during the process of learning. However, they need not be viewed as inherently opposing. As I will show, when interpreted through the conceptual lens of constructivist theory, they jointly illuminate different dimensions of the learning process.

Flow pertains to activities and processes in which the subject receives continuous positive feedback on their actions (cf. condition 2) and where their existing knowledge stands in productive relation to the challenges they encounter (cf. condition 3). However, flow theory was not originally developed as a theory of learning, nor was it originally intended for use within education specifically. As a result, flow theory does not account for the processes necessary to re-enter a state of flow once these conditions are no longer met beyond solely describing the conditions themselves. Shernoff and Csikszentmihalyi (2009) briefly comment on this:

Much like Vygotsky’s zone of proximal development zone, the level in which most learning occurs is just one step beyond skills one has already mastered. In this case, sufficient practice may be needed until the song is mastered. Once the song is played comfortably with relative ease, learning a new song at a higher level of challenge, causing one’s skill to increase yet again, can restart a cycle of fresh learning (p. 132).

While I acknowledge that this example closely resembles Vygotskij’s concept of the zone of proximal development, a fundamental distinction lies in the fact that Vygotskij, and scholars working within his tradition, offer concrete mechanisms to explain how learning occurs both within and beyond this zone, such as the process of internalization discussed above. Although the example presented by Shernoff and Csikszentmihalyi (2009) in the chapter *Flow in Schools: Cultivating Engaged Learners and Optimal Learning Environments* is indeed analogous (as they themselves note), they do not elaborate on how the necessary conditions for re-entering can be met once it is disrupted. Given the growing attention that flow theory receives within education, it would benefit from being conceptually linked to theories that more explicitly account for the cognitive processes underpinning learning.

Note that although limiting its scope, this does not entail a devaluation of flow theory’s potential within education, as the aforementioned scopes pertaining to student motivation and engagement have been widely utilized for both conceptual and empirical research with promising results (e.g., Reyes et al., 2012). In other words, the comprehensive work describing the experience of flow conducted by Csikszentmihalyi over the past five decades (consisting of over 8,000 interviews) does provide constructivist learning theories with important insights pertaining to the qualitatively lived experience of engaging in a process of learning, albeit solely concerning that which within the Piagetian school is termed assimilation. Sebastián et al. (2021) provide the following reflection on assimilation:

(…) when faced with a new object, every human being will tend to mobilize his previously equilibrated knowledge structures, in order to assimilate it into them. If the object is satisfactorily incorporated into the relatively stable sequences of action that has been set in motion in the subject, the sequence of actions will be completed without any difficulty of the subject (p. 238).

As evident, the concept of assimilation inherently involves a certain ease and stability, in that new knowledge objects can be integrated into existing cognitive structures without requiring the subject’s active, reflective engagement. Take a practical example: A 10-year-old student is introduced to the concept of fractions for the first time in mathematics. After a brief explanation, she immediately relates the idea to her prior experiences, such as dividing a chocolate bar or slicing a pizza. In doing so, she assigns meaning to the new term through embodied and familiar experiences. The concept of fractions thus becomes usable for her, allowing her to participate confidently in classroom discussions and solve problems. In this instance, assimilation has occurred smoothly, integrating the new concept into her existing knowledge structures. Given this foundation, the object-specific conditions for flow may all be present: (1) the goal of the activity is clear, (2) feedback is immediate, and (3) her skills and knowledge are in a productive relationship with the challenge at hand, and so on. If the more elusive subject-specific conditions are also met, such as (4) heightened attentiveness and concentration, (5) exclusion of irrelevant stimuli, (6) diminished self-consciousness, and (7) an altered sense of time, then the student can be deemed to be within a state of optimal experience, or flow.

However, as previously noted, not all learning situations unfold smoothly. Learners often encounter new information or objects of knowledge that appear deeply puzzling or confusing. Within flow theory, such situations would place the subject in a state of anxiety where the level of challenge surpasses their current capabilities and knowledge. This state of anxiety parallels Piaget’s concept of disequilibrium. When the subject becomes aware of this disequilibrium, which in the example above entails realizing that they do not understand what the concept of fractions signifies, a cognitive conflict emerges. At this point, the optimal conditions for flow are disrupted. From the perspective of learning as negation, this awareness of disequilibrium corresponds to what English (2012) describes as a pre-reflective experience of negativity, which represents a rupture in the subject’s habitual mode of being-in-the-world. In Buck’s terms, the cycles of anticipations (protentions) shaped by their ongoing primal impressions have been interrupted, as some sort of knowledge object did not appear to the subject as intuitively appropriate in the given situation. The learner is thus confronted with the fact that the object of learning lies beyond their current cognitive structures, resulting in heightened thematised self-awareness. Meyer-Drawe describes these moments as surprises wherein the learner experiences a “painful turn-around,” which within constructivist theories would be termed as enduring cognitive conflicts.

During cognitive conflicts, Piaget argues that the subject will inherently seek to re-establish equilibration by way of accommodation. Piaget links this to the concept of *homeorhesis*, which speaks to the autoregulatory mechanism that ensures that equilibrium is met (Friel, 2015). In situations of enduring cognitive conflicts, the process of accommodation is necessary to restore equilibrium. Accommodation, thereby, may be understood as a process through which the subject restores meaning when their usual ways of confronting the world are temporarily disrupted by the experience of cognitive conflict (Piaget, 1971). Considering that the disequilibrium, to a certain extent, needs to be *experienced* as meaningful for the subject for the cognitive conflict to spark motivation for the process of learning, processes of reflection and meaning-making are central. As is also argued by Sánchez and Sebastián (2024):

(…) reflection is based on the experience of doubt, challenge, and uncertainty, and guides the subject towards achieving a specific goal (…) the sense of uncertainty and relationality between the learner and the learning situation as an unfinished process would motivate a certain awareness of the learning process as it unfolds (p. 242).

This overlaps nicely with the descriptions of those working with learning as negation, namely the uncertainty faced when interacting with a knowledge object that, in constructivist terms, cannot be assimilated into existing knowledge structures.

According to the insights provided through learning as negation, the processes within constructivist theories that are termed accommodation are characterized by a thematized self-awareness and reflection. This themstised self-awareness can, on Buck’s account, only be productively handled in processes of reflection, conceptualized through Hegel’s conceptual framework of negation within the dialectic method. This, again, aligns with the recent conceptual advances seeking to integrate Vygotskij’s conceptualisation of internalization with Piaget’s concept of equilibration. Here, internalization is conceptualized as a dialectic process between a subject’s psychological processes and their meeting with the world around them [Álvarez-Espinoza and Sebastián Balmaceda, 2018; Castorina and Baquero, 2005, cited in Sebastián et al. (2021)].

This, in turn, signifies the importance of the concept of reflection, as reflection can serve as the mediator that connects the theories that explicate the experience of learning with those that more closely refer to the psychological processes that underpin these experiences. The reason why reflection holds such significance for this purpose is not only limited to its widespread application in research pertaining to the process of learning (e.g., McCabe and Thejll-Madsen, 2020) but also pertaining to its unique so-called conceptual contradiction pointed out by Mälkki and Green (2018): *While the automatic filtering provided by meaning perspective provides us the very tools to make sense of our experience, reflection aims to interrupt this very tendency.* (p. 29). However, the authors go on to construe a contradictory tension between subjects’ search of meaning, on the one hand, and reflection’s inherent interruption of the sensemaking of experience. While I agree with the former, reflection does, in fact, break with the continuity of experience (understood here through Husserl’s triadic structure). The latter postulates that making sense of experiences and reflecting upon them in a competing relationship is not convincing, considering the equilibration process. Rather, reflection within a constructivist outlook on learning is more aptly conceptualized as (1) a prerequisite for the emergence of cognitive conflict, seeing that this is dependent on the subject becoming aware of an enduring disequilibrium, as well as (2) the path through which the process of accommodation can occur (i.e., re-establishing equilibrium.). However, for the second point to align with recent advancements toward a general theory of learning (Sebastián et al., 2021), reflection needs to be positioned within the framework of the zone of proximal development and internalization; meaning that reflection in the latter point must account for social processes. This perspective, however, is not necessarily controversial, as the notion of incorporating social and interpersonal aspects in conceptualizations of reflection is already well established (e.g., Green, 2016).

Furthermore, as its etymology suggests; *re*-flection entails the action or process of shedding light back toward that which was its initial source, i.e., reflecting on the experiences or objects of knowledge past. This aspect of reflection is highly important within learning as negation, as realigning and re-interpreting previous experiences in light of the newly faced challenges serve as central to making sense of a negated phenomenon. What the constructivist tradition offers the concept of reflection in this context is unpacking reflection in such a way that it functions as an avenue toward realigning the learner’s perception of their previous experiences so that the current knowledge structures can make sense of their encounters with the world in the present (through the process of accommodation). Within Husserlian terminology as applied by Buck: In a meeting with a negated anticipation (protention), the break in experience from the primal impression entails an examination, i.e., reflection upon the negated protention, as well as a re-evaluation of past experiences, so that they can align with the newly learned knowledge objects (Buck, 2019; Meyer-Drawe, 2018, 2019).

In summary, flow theory and learning as negation contribute to a wider conceptual apparatus that illuminates the experiential dimensions and activity of consciousness while engaging within those processes that contemporary learning theories commonly regard as central: (1) experiences that appear intuitive to the subject, (2) the emergence of sustained cognitive conflict, and (3) the cognitive processes involved in resolving such conflicts.

## Concluding remarks

Does the upshot of this discussion suggest that all learners are caught in a constant oscillation between optimal experience and painful turn-arounds? Well, not necessarily. There is also a need for conceptualizations that account for the more nuanced, less dramatic experiences that lie between these two poles. Some scholars have already attended to these experiences, for instance, Mälkki and Green (2018) introduce the notion of *edge-emotions*, which arise at “the edges of our comfort zones when our meaning perspectives become questioned” (p. 30). Considering this, Fuchs’ framework can contribute to differentiating degrees of experienced disequilibrium. The protentional cone (Fuchs, 2022) illustrates how individuals encounter the world within the boundaries of what is probable (akin to assimilation). When a primal impression deviates from this range of probability, however, disequilibrium occurs. The proximity and intensity of this deviation in relation to the initial primal impression influence how much it encumbers the subject’s active consciousness, which in turn determines whether it is experienced as an enduring cognitive conflict. If the primal impression is immediately faced with an improbable outcome (a negation of protention, in Buck’s terms), it is more likely to generate sustained cognitive conflict than if the deviation emerges more distantly. However, these processes are not only temporally situated but also thematically situated depending on the previous convictions of the subject. In other words, even if a negation occurs with temporal distance from the initial impression, it may still produce enduring conflict if it contradicts the subject’s previous experiences in a socially or biographically meaningful way (cf. Bourgeois, 2011; Sánchez and Sebastián, 2024).

**Figure 3:**
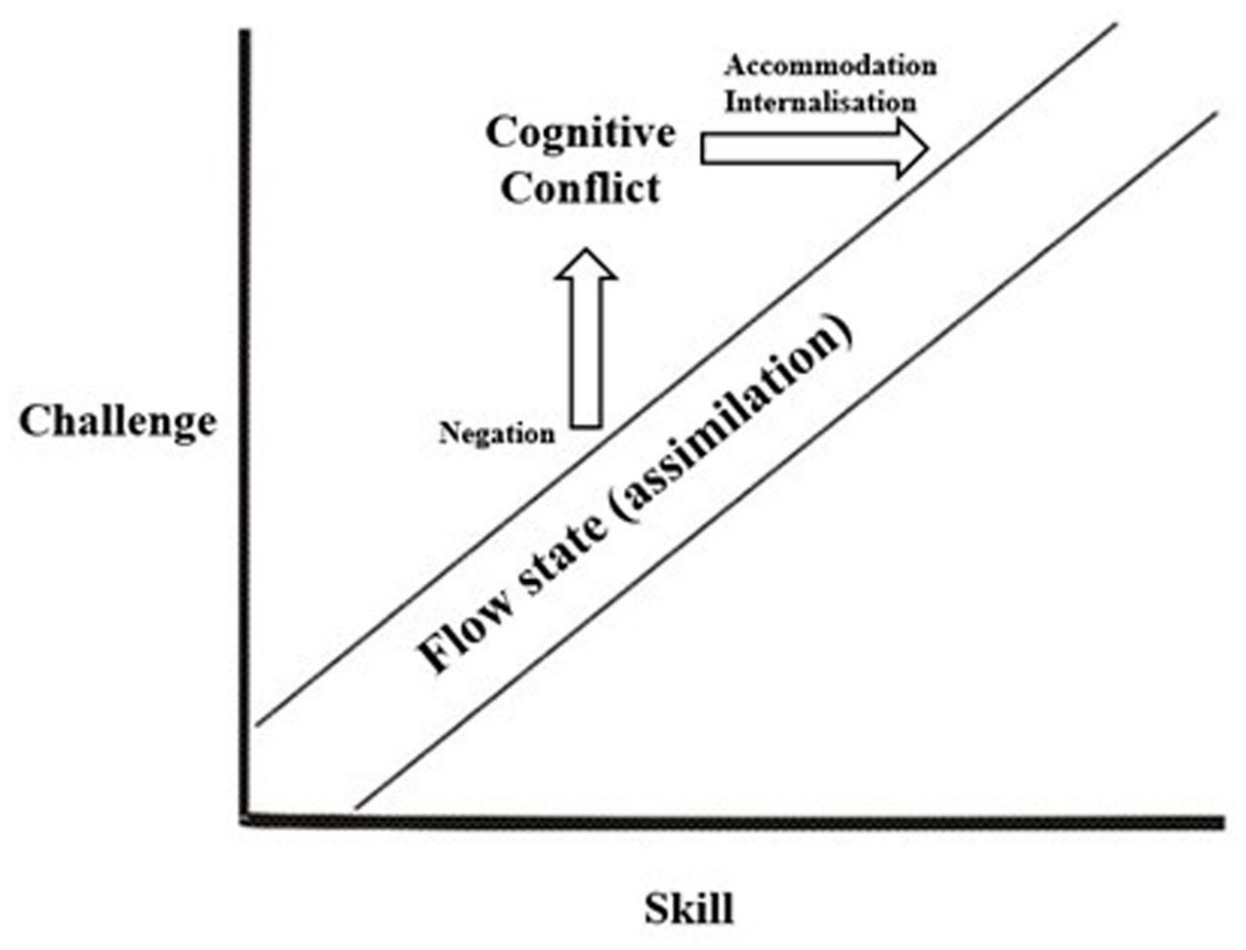
The experience of learning—derived from Csikszentmihalyi (1990), supplied with terminology from Piaget, Vygostkij and Buck.

These insights are integrated into Figure 3. Here, the distance on the Y-axis from the flow zone, where assimilation occurs effortlessly, caused by the negated protention, reflects both the extent to which previous experience must be restructured, as well as the degree of scaffolding needed for internalization (represented by the length of the arrows).

The more transformative the experience is, the “further up” will the subject re-enter the flow zone. This implies that the learner’s cognitive structures have evolved, enabling the assimilation of more complex knowledge objects, thus characterizing a transformative learning experience. This aligns with Sebastián et al.’s (2021) reading of Piaget, where the authors posit that:

The new structure that emerges from the transformation of the initial structure is capable not only of returning the subject to the form of equilibration that he was carrying out before the cognitive conflict but to an even more stable and complex one in which the types of objects that caused the cognitive conflict can be assimilated, and also other types of objects, typically of a higher logical level (p. 239).

Note that visualizations of the flow channel that contains different plot points either within the field of anxiety or boredom is not at all a novel addition in itself, as many such alternatives have already been made see (e.g., Csikszentmihalyi and Rathunde, 2014). However, although integrating plot points, like I have done here, has been done previously, explicit investigations into *how* this movement occurs have not. The novelty of the theoretical dialogue between the different facets of scholarship within this study lies namely in the unique interplay between them. One the one hand, learning as negation and flow theory provides rigorous and detailed descriptions about the structure of consciousness and the experience of effortless mastery during the learning process, respectively. However, scholars working with either perspective have seldom engaged in discerning the cognitive processes that underpin these experiences. On the contrary, studies that explicitly account for the cognitive processes that underpin learning are calling for more research into the role of emotions, affectivity, and experience in the process of learning. As such, this study serves as one step in bridging the different theoretical perspectives provided on the process of learning and the learners’ experiences therein. In conclusion, each strand of scholarship has contributed to a more mature and nuanced account of the learning process through their integration. This account builds on existing literature pertaining to a general theory of learning, offering a perspective that illuminates the lived experience of learning while also addressing the cognitive processes that underpin it.

## Data Availability

The original contributions presented in the study are included in the article/supplementary material, further inquiries can be directed to the corresponding author.

## Footnotes

^1^Note, I do not intend to present these theories in the depth required for a comprehensive understanding of their full scope. Rather, I offer a general outline, limited to the concepts and ideas relevant for the analyses that follow.

^2^Note that there are also figures where there are demarcations of different types of boredom and anxiety. Although highly useful in certain settings, such visualizations would not contribute any meaningful insights toward my discussion in this article.
